# Supplementary material for: Testosterone reduces uterine contractions in vivo: Evidence for non-genomic action in rats
Source: PLoS One. 2026 May 5;21(5):e0348344. doi: 10.1371/journal.pone.0348344 (PMC13143053; doi:10.1371/journal.pone.0348344)
Supplement: Attachment — Submitted filename: Responses to Reviewers.docx [file pone.0348344.s009.docx]

**RESPONSE TO REVIEWERS**

The authors thank the Editor and the Reviewers for their useful questions and remarks which have contributed considerably to improving the quality of our manuscript. Our answers are given below.

**Responses to the Editor**

***To comply with PLOS One submissions requirements, in your Methods section, please provide additional information regarding the experiments involving animals and ensure you have included details on (1) methods of sacrifice, (2) methods of anesthesia and/or analgesia, and (3) efforts to alleviate suffering.***

For in vivo experiments, animals were anesthetized with a combination of ketamine and xylazine (36+4 mg/kg), as indicated in the first version of the manuscript, and then euthanized with a ten-fold dose (ketamine and xylazine 360+40 mg/kg). For in vitro experiments, the animals were euthanized in a CO_2_ chamber with gradually increasing carbon dioxide concentrations. The ways of euthanasia of the animals are also indicated in the revised version of the manuscript.

***Thank you for stating the following financial disclosure: “Project No. TKP2021-EGA-32 was implemented with the support provided by the Ministry of Innovation and Technology of Hungary from the National Research, Development, and Innovation Fund, financed under the TKP2021-EGA funding scheme. The work was supported by the Stipendium Hungaricum Scholarship, University of Szeged, Hungary and by the Research Fund of Albert Szent-Györgyi Medical School, University of Szeged, Hungary. The work was also funded by the Research Fund of Albert Szent-Györgyi Medical School, University of Szeged, Hungary and University of Szeged Open Access Fund (Grant number 8261)” Please state what role the funders took in the study. If the funders had no role, please state: "The funders had no role in study design, data collection and analysis, decision to publish, or preparation of the manuscript." If this statement is not correct you must amend it as needed. Please include this amended Role of Funder statement in your cover letter; we will change the online submission form on your behalf.***

We made the following statement: “The funders had no role in study design, data collection, analysis, decision to publish, or preparation of the manuscript.” This statement is included indicated in the revised manuscript.

***We note that your Data Availability Statement is currently as follows: All relevant data are within the manuscript and its Supporting Information files. Please confirm at this time whether or not your submission contains all raw data required to replicate the results of your study. Authors must share the “minimal data set” for their submission.***

All raw data necessary to replicate the results have been included as supplementary information in separated excel files.

Editor Comments

1. ***I am so sorry, but I could not understand the meaning of Dose 1 and Dose 2 in Figure 2.***

Thank you for your comment. In this study, we examined the possible independent uterine effect of the administered solvent. The doses refer to 100 microliters of the administered solvent, which was administered twice when the antagonist was used. For ease of understanding, we have modified the notation to **First** and **Second** administration in the revised version of the manuscript.

2. ***Please provide in-brief description of experiment in the legend of figures and title of Tables as well as sample size and Data (Mean +/- SD).***

The requested descriptions have been provided in the revised version of the manuscript.

3. ***Please show statistical differences for T levels between pregnant and non-pregnant rats in Figure 4; It could be useful to indicate the time of T injection by an arrow.***

The statistical differences were presented, and the timing of testosterone injections was also added to the figure (**Figure 5** in the revised manuscript) and discussed in the revised manuscript.

4. ***I would suggest to include the baseline HPPD level for comparisons shown in Figure 7***

The NP and P controls represent baselines (**Figure 9** of the revised manuscript), as presented in the first version of the manuscript.

5. ***Sensitivity and specificity of T and HPPD ELISA kits are missing; hence the detection ranges were provided.***

The sensitivity of the ELISA kits is 18.75 pg/ml for both T and HPPD. Both are specific for testosterone or HPPD only, and there is no apparent cross-reactivities. This information has been included in the Methods section of the revised manuscript.

6. ***Please provide the method to avoid the blood coagulation to obtain the plasma.***

A 1 ml blood sample was taken from the tail vein before and after dosing (0, 5, 15, 30, 60, 120, 240, and 480 minutes) into tubes containing K_2_EDTA (1 mg/tube) (BD Microtainer, Thermo Fisher Scientific Inc., Budapest, Hungary), then centrifuged (1700× g, 10 minutes, 4 °C) to isolate the plasma. This methodological description was also included in the revised version of the manuscript.

7. ***Please provide a single paragraph abstract***

The single paragraph abstract was provided in the revised version of manuscript.

8. ***Please add significance of the study at the beginning of abstract***

We modified the significance of the study based on the reviewers' comments and indicated this in the summary of the revised version of the manuscript.

**Responses to Reviewer #1**

1. ***The Abstract does not report any numerical information regarding the study population (number of animals included, size of the study and control groups, allocation, etc.). These details are essential for transparency and should be included to provide readers with a clear understanding of the sample structure.***

The study population data were added to the abstract in the revised version of the manuscript. Female rats received only testosterone (3/10/30/100/300 mg/kg ip), flutamide (100 mg/kg ip), solvent (DMSO+Macrogol 25%+75%, 1 ml/kg ip) or normal saline (1 ml/kg) (n=5-8/group, 160-220 g). To measure testosterone and HPPD plasma levels, female rats weighed 200-220 g (n=4-8/group).

2. ***The Methods section does not describe whether any criteria were used for selecting the male rats. It is important to specify age, weight, health status, breeding conditions, and any exclusion criteria, as these factors may influence hormonal and physiological responses.***

The study was conducted in female rats (160-220 g) to determine the effect of testosterone on the contractile capacity of non-pregnant and pregnant uterus. Pregnant animals were used on the last day of pregnancy (day 22 of pregnancy), while non-pregnant animals were selected based on the estrus phase of the estrous cycle to minimize differences caused by hormonal effects. Male rats were used for mating only and weighed between 240 and 260 g. All animals were healthy, with no diseases or adverse symptoms observed. Body weight and the term "healthy" were incorporated into the revised manuscript.

3. ***Confounding Effect of General Anesthesia on Uterine Contractility***
A. ***A major potential confounder is the assessment of uterine contractility during general anesthesia, as anesthetic agents may alter smooth muscle function and hormonal responsiveness. The authors should discuss the known impact of general anesthesia on uterine contractility and justify its use.***

It is known that general anesthetics relax the smooth muscles of the uterus, so we chose a combination of ketamine and xylazine, which causes moderate relaxation compared to other general anesthetics. Since we conducted a self-controlled experiment, any initial relaxation caused by the general anesthetic was part of the control phase in all cases.

B. ***Furthermore, it should be clarified why regional or local anesthesia was not considered, or whether such an approach would have been feasible in this experimental model.***

General anesthesia is required to keep the animal’s unconscious to avoid muscle movements and changes in body position that would significantly interfere with the measurement of uterine contractions. Epidural or spinal or regional local anesthesia cannot be used because awake rats are unable to cooperate during measurement.

4. ***Rationale for Intraperitoneal Administration of Testosterone***
A. ***The rationale for administering testosterone intraperitoneally is not explained. The authors should clarify why this route was chosen over others (e.g., intravenous, subcutaneous), describe its pharmacokinetic implications.***

Testosterone is lipid-soluble (insoluble in water), so we dissolved it in a combination of DMSO and macrogol. Due to the toxicity of such solutions (e.g., hemolysis, risk of precipitation), they are not suitable for intravenous administration, so we chose the second fastest-acting method of administration, which was intraperitoneal administration.

B. ***Discuss whether this method holds translational value for potential human studies. Without this clarification, the clinical relevance of the findings remains uncertain.***

Intraperitoneal administration of testosterone has no direct translational significance, as this type of administration is not used in clinical practice. However, our study provides the first evidence of the uterine-relaxing effect of testosterone in vivo during pregnancy. These results may therefore justify the development of new, more water-soluble testosterone analogues that allow for dosing methods that are already applicable in clinical practice.

5. ***Results – Missing Numbers of Animals Included***
A. ***The Results section does not report how many rats were actually included in the study or how many were allocated to each group. This information is fundamental for interpreting the statistical power and the reliability of the findings. The exact number of animals per group must be added.***

The number of rats per group is indicated in the revised version of the manuscript.

6. ***The concluding statement suggesting that testosterone should be used for the treatment of threatened preterm labor is inappropriate and not supported by the presented data. The study was conducted in rats, under experimental conditions, with unclear translational applicability. Such a statement should be removed, and the conclusion should be limited to the observed experimental findings without extrapolating to clinical practice.***

Thank you for your comment. We agree with the reviewer that our translation conclusion in the first version of the manuscript was rather speculative, so we have modified our presumed translation significance to the following: " Testosterone or its analogues may be good candidates for further preclinical and clinical studies for the potential treatment of conditions associated with uterine hyperactivity."

**Responses to Reviewer #2**:

1. ***The central hypothesis that testosterone acutely reduces uterine contractility in vivo is biologically plausible, but the mechanistic rationale is insufficiently developed, particularly regarding receptor-mediated vs non-genomic effects.***

We previously published an in vitro study in which testosterone induced a rapid non-genomic relaxing effect in the uterine smooth muscle of 22-day pregnant rats. The non-genomic effect was demonstrated by the ineffectiveness of the RNA transcription inhibitor actinomycin D and the protein synthesis inhibitor cycloheximide, which did not modify the effect of testosterone. [doi: 10.1016/j.lfs.2020.118584].

Furthermore, in our present study, we demonstrated that the 30-minute effect of testosterone did not induce any genomic response in vivo, as inferred from unchanged plasma 4-HPPD levels. The uterine relaxant effect of testosterone was not altered in the presence of an androgen receptor blocker (flutamide). Furthermore, during manuscript revision, we performed *in vitro* measurements to confirm the most likely non-genomic mechanism of action of testosterone via calcium channels. Testosterone (10^-4^ M) blocked calcium chloride-induced contractions in a calcium-free environment in a manner like nifedipine (10^-7^ M), strongly suggesting a role for calcium blockade in the non-genomic effects of testosterone. These new *in vitro* results have been incorporated into the revised version of the manuscript.

2. ***The manuscript does not clearly distinguish whether observed effects are direct uterine actions or secondary to systemic endocrine or autonomic modulation.***

The effect of testosterone is most likely not related to secondary endocrine effects, as such endocrine effects take longer than the 30-minute time interval used in our studies. Furthermore, testosterone exerts a similar direct effect on the uterus in vitro, as we observed in our previous study [https://doi.org/10.1016/j.lfs.2020.118584].

We agree with the reviewer that testosterone may have autonomous effects on uterine contractions via calcium channels and G protein-coupled membrane receptors, but these targets are in smooth muscle cells, so it seems very difficult to clearly distinguish between the autonomic and uterine smooth muscle effects of testosterone, especially *in vivo*. Nevertheless, the reviewer's idea is excellent, and we will take it into account in our future studies.

3. ***Experimental Design:***
A. ***Estrous cycle control is inadequately addressed. Given the profound influence of cycle stage on uterine contractility and steroid responsiveness, failure to rigorously synchronize or stratify animals represents a major biological confounder.***

As described in the first version, all experiments on non-pregnant rats were performed during the estrus phase, and the rats were selected based on vaginal impedance using the Estrus Cycle Monitor (IM-01, MSB-MET Ltd., Balatonfüred, Hungary). For non-pregnant experiments, rats with vaginal impedance between 4.5 and 7.5 kΩ on the day of the experiment (estrus phase interval) were selected. This type of selection of non-pregnant animals is a well-established and widely accepted method for avoiding endocrine disrupting factors.

B. ***The choice of testosterone dose and route of administration lacks sufficient justification in terms of physiological relevance. It is unclear whether serum levels achieved correspond to: physiological female exposure, pathological hyperandrogenism, or pharmacologic supraphysiological conditions.***

The testosterone dosage range was selected based on the literature on intraperitoneal systemic testosterone administration in male rats [https://doi.org/10. 1016/j.bbr.2014.01.013], as no data were available on testosterone administration in female rats, especially during pregnancy. Testosterone administration significantly increased plasma testosterone levels in both non-pregnant and pregnant rats, resulting in a 10-30-fold increase after the highest dose (300 mg/kg). These serum levels are achievable with exogenous testosterone administration. This single testosterone administration was not intended to model any physiological, pathophysiological, or supra-physiological condition, but only to measure the dose-dependent uterine relaxing effect of testosterone. Our animal model is not related to any endocrine disease.

C. ***The timing of outcome assessment relative to hormone administration is not clearly justified, limiting interpretation of genomic vs non-genomic effects.***

As we wrote in the first version of the manuscript, there is solid evidence supporting the link between timing and non-genomic effects, so we chose the time for evaluating the results (less than 30 minutes) to be insufficient for a genomic response to develop, and the effects obtained could be attributed to a non-genomic effect [https://doi.org/10.1155/2020/8849641, https://doi.org/10.1016/j.steroids.2019.108509]. The ineffectiveness of the testosterone antagonist flutamide also confirms that we obtained non-genomic responses.

4. ***Outcome Measures:***
A. ***Uterine contractility is treated as a unitary endpoint, but: Frequency, amplitude, and coordination of contractions are not consistently analyzed or discussed as distinct physiological variables.***

We chose AUC-based evaluation to determine contraction changes. Since the AUC change includes both frequency and amplitude changes, it provides a complex but simple parameter for characterizing uterine contractions: if the frequency or amplitude decreases, it means that uterine activity has decreased, which is sensitively detected by the AUC analysis. The strain gauge method cannot be used to measure the coordination of contractions because this method cannot detect electrical signals.

B. ***No direct assessment of myometrial androgen receptor expression or signaling is provided, weakening causal inference.***

We disagree with the reviewer regarding the lack of evaluation of androgen receptor signaling in the myometrium, as our experiments were performed in the presence of an androgen receptor antagonist (flutamide), which had no effect on testosterone function, suggesting that androgen receptors play no role in the effects measured. It is true that we do not know the expression of androgen receptors in the myometrium, but this is not relevant at this stage of our non-genomic experiments. Mapping the androgen receptor status of the uterus may indeed be important in relation to possible long-term adverse effects, but this was beyond the scope of the study.

5. ***Controls and Comparators:***
A. ***The absence (or limited use) of: estrogen-only controls, androgen receptor antagonists, or ovariectomized replacement models significantly limits mechanistic interpretation.***

Since the non-pregnant rats were all in the same phase of the estrous cycle, estrogen control or removal of the ovaries would not add much value. Furthermore, the effect of endogenous estrogen is certainly mostly genomic and should have a very similar effect on uterine contractions at the same phase of the estrous cycle. Based on these considerations, we do not see any limitations in the absence of the interventions mentioned by the reviewer. Furthermore, these interventions are not applicable during pregnancy.

B. ***Vehicle controls are insufficiently discussed, particularly regarding stress and injection effects on uterine activity.***

Surgical intervention and intraperitoneal injection do not have a significant effect on uterine contractions, as all procedures were performed under general anesthesia, as mentioned in the first version of the manuscript. In addition, all animals underwent identical surgical procedures and dosing regimens, so the presumed stress factor should also be present in animals treated with testosterone. These considerations are also considered in the discussion of the revised manuscript.

6. ***Interpretation of Results:***
A. ***The discussion overstates causality, implying direct androgenic suppression of uterine contractility without adequately excluding indirect pathways.***

Thank you for this interesting question. In the revised manuscript, we show that testosterone blocks calcium-induced contractions in vitro, an effect that was previously suspected based on its ability to reduce intracellular calcium levels [https://doi.org/10.1095/biolreprod.104.036954, doi: 10.1152/ajpheart.00753.2009]. Further studies are needed to determine whether the uterine relaxing effect of testosterone is exclusively related to the inhibition of calcium channels or whether steroid membrane receptors also play a role in relaxation. It is likely that both mechanisms are involved. These considerations are also discussed in the revised manuscript.

B. ***Translational implications for human reproductive physiology are speculative and insufficiently qualified, given species-specific differences in myometrial steroid responsiveness.***

There are similarities in uterine hormone responsiveness between rats and human as many *in vitro* studies conducted in human myometrium and exhibited the similar response of androgens as in rats [<https://doi.org/10.1095/biolreprod.104.036954>], however we agree with the reviewer that our translation conclusion in the first version of the manuscript was rather speculative, so we have modified our presumed translation significance to the following: " Testosterone or its analogues may be good candidates for further preclinical and clinical studies for the potential treatment of conditions associated with uterine hyperactivity."

7. ***Statistical Considerations:***
A. ***Sample size justification is weak or absent.***

The minimum number of experimental animals required for evaluable final results in *in vivo* experiments was calculated using version 3.1.2 of the Power and Sample Size program with the following parameters: α= 0.0125 (type I error); 1-β=0.8 test power; m=1 (considering the number of control and treated individuals to be equal in each group); effect size=1.66, which is the ratio of the difference between the means of the treatment groups and the variance of the data. As a result of the estimation, we plan to conduct the experiments with a final sample size of n=4-8 animals/group. Based on our decades of experience with *in vitro* experiments, at least 6 animals per group are required to accurately measure the relaxation effect, considering individual differences in contractility observed in tissue sensitivity.

B. ***Repeated-measures structure (if present) is not clearly accounted for, raising concerns about pseudoreplication.***

We did not perform repeated measurements or mixed-effects analyses. In the ANOVA analysis we performed, there is no pairing; all data come from different individuals.

C. ***Variability within treatment groups suggests biological heterogeneity that is not explored.***

Biological heterogeneity is significant when both sexes are included in the study, or when animals are kept under different conditions, or when data from multiple centers need to be compared. In our case, none of these apply, as we studied female rats that were kept in the same animal facility and measured their responses in the same laboratory. The variability within the treatment groups is not high, considering that individual responses in *in vivo* experiments are never as similar as in *in vitro* experiments. The differences in responses between the treated *in vivo* groups are not large or unusual, so we saw no reason to investigate the presumed biological heterogeneity. Additionally, we checked the coefficients of variation during the ANOVA analysis, and the values were within the acceptable range.
