# Supplementary material for: Testosterone reduces uterine contractions in vivo: Evidence for non-genomic action in rats
Source: PLoS One. 2026 May 5;21(5):e0348344. doi: 10.1371/journal.pone.0348344 (PMC13143053; doi:10.1371/journal.pone.0348344)
Supplement: Attachment — Submitted filename: Responses_to_Reviewers_auresp_2.docx [file pone.0348344.s010.docx]

**Editor Comments:**

***1- As AR was not been assessed, I would suggest to remove “evidence for non-genomic action” from the title. Use of Flu as an antagonist for AR is not enough.***

Thank you for your comment, but we believe it is better to keep the title as it is. We demonstrated that 30 minutes of testosterone exposure did not elicit any genomic response in vivo, as inferred from unchanged plasma 4-HPPD levels, so we did not rely solely on the androgen antagonist-resistant effect. In addition, we previously published an in vitro study in which testosterone elicited a rapid, non-genomic relaxing effect in the uterine smooth muscle of 22-day-old pregnant rats. The non-genomic effect was demonstrated by the ineffectiveness of the RNA transcription inhibitor actinomycin D and the protein synthesis inhibitor cycloheximide, which did not modify the effect of testosterone. [doi: 10.1016/j.lfs.2020.118584].

***2- I would suggest to add a schematic cartoon summarizing T inhibition of uterine contraction. It could be cited in Conclusion as Fig. 10.***

Thank you for this thoughtful comment; it was considered, and Fig 10 was added in the revised version of the manuscript.

***3- It would be great if you edit the English of MS. There are many errors.***

Thank you for this advice, it was considered and the English of the manuscript was edited in the revised version.

***4- Please add data availability statement.***

We added it in the revised version of the manuscript.

***5- I would suggest to use T as an abbreviation for testosterone after expanding in its location.***

The abbreviations were considered throughout the manuscript in the revised version

6. All other comments were considered and edited in the revised version of the manuscript.
