# Supplementary material for: Testosterone reduces uterine contractions in vivo: Evidence for non-genomic action in rats
Source: PLoS One. 2026 May 5;21(5):e0348344. doi: 10.1371/journal.pone.0348344 (PMC13143053; doi:10.1371/journal.pone.0348344)
Supplement: Attachment — Submitted filename: Responses_to_Reviewers_auresp_3.docx [file pone.0348344.s011.docx]

Journal Requirements:

1. ***If the reviewer comments include a recommendation to cite specific previously published works, please review and evaluate these publications to determine whether they are relevant and should be cited. There is no requirement to cite these works unless the editor has indicated otherwise.***

There was no such comment.

2. ***Please review your reference list to ensure that it is complete and correct. If you have cited papers that have been retracted, please include the rationale for doing so in the manuscript text, or remove these references and replace them with relevant current references. Any changes to the reference list should be mentioned in the rebuttal letter that accompanies your revised manuscript. If you need to cite a retracted article, indicate the article’s retracted status in the References list and also include a citation and full reference for the retraction notice.***

The reference list is complete; we do not cite any retracted article.

Additional Editor Comments:

1- ***Supplementary materials: I do not know why S1 is titled Fig 3? or S2 is Fig4? Usually, S1 should be Fig 1, S2 should be Fig 2, S3 Table 1, S4 Table 2, S4 Fig 3 and so on.***

Thank you for the comment. The fact is that Fig 1 and Fig 2 do not have any data to put in supplementary files (representative or schematic figures). Thus, the first figure that has supplementary material is Fig 3.

2- ***It is not acceptable to respond "All other comments were considered and edited in the revised version of the manuscript". The letter of response should answer all comments point-by-point.***

***L20: This study aimed to investigate the non-genomic effect of testosterone (T) on uterine muscle contractility in non-pregnant and 22-day pregnant rats, in vivo.***

Thank you, we accepted the recommendation and the sentence is involved in the revised version of the manuscript as “This study aimed to investigate the non-genomic effect of testosterone (T) on uterine muscle contractility in non-pregnant and 22^nd^ day pregnant rats, *in vivo*”.

***L21: Circulating T levels were measured withing 8 hours after a single T administration (10 mg/kg ip) by ELISA.***

Thank you, we accepted the recommendation and the sentence is involved in the revised version of the manuscript as “Circulating T levels were measured within 8 hours after a single intraperitoneal (ip) administration of T (10 mg/kg) by ELISA”.

***L36: Please re-write: Testosterone plasma levels showed dose-dependency as well.***

Thank you, we accepted the recommendation and the sentence is involved in the revised version of the manuscript as “T plasma levels were proportional with the administered doses”.

***L37: remained unchanged in both 22-day non-pregnant and pregnant rats  after 30 min of T administration***

Thank you, we accepted the recommendation and the sentence is involved in the revised version of the manuscript as “remained unchanged in both non-pregnant and 22^nd^ day pregnant rats after 30 min of T administration”

***L38: A single dose T induces***

Thank you, we accepted the recommendation and the sentence is involved in the revised version of the manuscript as “A single dose T induces…”

***Testosterone is the most important hormone of the androgen family in both males and***

***L55: … females. In males, T is mainly produced in the Leydig cells of the testes (8,10).***

Testosterone (T) is the most important hormone in the androgen family in both males and Thank you, we accepted the recommendation and the sentence is involved in the revised version of the manuscript as “females, in males produced mainly in the Leydig cells of the testes”

***L57: If you provide values for rats, would be perfect and meaningful with your experimental model.***

Thank you, we accepted the recommendation and the sentence is involved in the revised version of the manuscript as “Plasma T levels in males are higher than in females (2,7,11) or in pregnant women (4,11)”

***L97: Please indicate the minimum number of animals derived from calculation***

Thank you, we accepted the recommendation and the sentence is involved in the revised version of the manuscript as follow “The minimum number of animals per group was 4”

***L116: T, flutamide, and nifedipine were purchased from Sigma-Aldrich (Budapest, Hungary), DMSO was purchased from Fisher Scientific (Loughborough, UK), and Macrogol 400  was purchased from MAGIlab Ltd. (Budapest, Hungary). The rat T and hydroxyphenylpyruvate dioxygenase (HPPD) ELISA kits were purchased from Wuhan Fine Biotech Co. Ltd. (Wuhan, China).***

Thank you, we accepted the recommendation and the sentence is involved in the revised version of the manuscript as “T, flutamide, and nifedipine were purchased from Sigma-Aldrich (Budapest, Hungary), DMSO was from Fisher Scientific (Loughborough, UK), Macrogol 400 was purchased from MAGIlab Ltd. (Budapest, Hungary), while Rat T and 4-HPPD ELISA kits were delivered by Wuhan Fine Biotech Co., Ltd. (Wuhan, China)”.

***L121: 2.4. Plasma T assessment***

Thank you, we accepted the recommendation, the 4-HPPD method was merged with testosterone, and the subtitle is involved in the revised version of the manuscript as “Plasma T and 4-HPPD assessment”

***L122: intraperitoneal  administration (ip)***

Thank you, we accepted the recommendation and the sentence is involved in the revised version of the manuscript as “intraperitoneal (ip) administration”

***L125: It may be omitted: “it is specific for testosterone only and there are no apparent cross-reactivities.“***

Thank you, we accepted the recommendation and the sentence was omitted.

*L129: for the plasma*

Thank you, we accepted the recommendation and the sentence is involved in the revised version of the manuscript as “…for the plasma”

***L159: Similar to L122 (aforementioned comment”***

Thank you, we accepted the recommendation and the sentence is involved in the revised version of the manuscript as “..ip administration”

***L160-165: It was aforementioned for T assessment. I would suggest to merge T and HPPD assessments to avoid redundancy.***

Thank you, we accepted the recommendation and the sentences are involved in the revised version of the manuscript as “Additionally, 4-HPPD levels were measured before (baseline) and 30-minutes after the administration of the T doses (100 or 300 mg/kg) for both non-pregnant estrus rats and 22^nd^ day pregnant (n=6-8/group) using a commercial rat 4-HPPD Enzyme Immunoassay Kit (Wuhan Fine Biotech Co., Ltd., China), with a detection range between 31.25-2000 pg/ml and sensitivity of 18.75 pg/ml”.

***L193: ip administration of ketamine + xylazine (36 + 4 mg/kg in 20 ml solution) in a dose of 5 ml/kg.***

Thank you, we accepted the recommendation and the sentence is involved in the revised version of the manuscript as “ip administration of ketamine + xylazine (36+4 mg/kg in 20 ml solution) in a dose of 5 ml/kg”.

***L196: into 4 experimental groups, including (1) solvent control, (2) T, (3) T + flutamide, and (4) absolute control (n=5-8 per group).***

Thank you, we accepted the recommendation and the sentence is involved in the revised version of the manuscript as “…..into 4 experimental groups including (1) solvent control, (2) T, (3) T + flutamide, (4) absolute control (n=5-8 per group)”.

***L198: ip dose of***

Thank you, we accepted the recommendation and the sentence is involved in the revised version of the manuscript as “..ip dose of”.

***L199: (100 mg/kg). The solvent***

Thank you, we accepted the recommendation and the sentence is involved in the revised version of the manuscript as “(100 mg/kg). The solvent..”

***L200: l/kg ip). The …. Physiological saline***

Thank you, we accepted the recommendation and the sentence is involved in the revised version of the manuscript as “The absolute control group received physiological saline”

***L134, 153, 164, 206: If all values are mean +/- SD; please avoid reducnancy and indicate it in the “Statistical analysis section”.***

Thank you, we accepted the recommendation they were deleted and added to the statistical analysis section, the sentence is involved in the revised version of the manuscript as “All data are expressed as means ± standard deviation (SD)”.

***Figure 2: ip should be in small letters.***

Thank you, we accepted the recommendation and the “ip” abbreviation are in small letter in the revised figure in the revised version of the manuscript.

***Figure 4: Please clarify whether T1 and T2 referrers to Time 1 and Time 2, respectively. I would suggest a revision for x-axis of panels C and D.***

Thank you, we accepted the recommendation and it is “Time 1” and “Time 2” in the revised version of the manuscript.

***L276: Fig. 8***

Thank you, we accepted the recommendation and it is involved in the revised version of the manuscript as “Fig. 8”

***L282: Please expand Emax and ED50  in the title of Table 2***

Thank you, we accept the recommendation and the table was corrected and is involved in the revised version of the manuscript as:

| Parameter | Non-pregnant rats | | 22^nd^ day pregnant rats | |
| --- | --- | --- | --- | --- |
|  | T | T+Flut | T | T+Flut |
| E_max_ (%) | 67.5 ± 5.9 | 72.9 ± 7.2 | 44.4 ± 5.9^**^ | 42.8 ± 6.9^**^ |
| ED_50_ (mg/kg) | 11.7 ± 1.9 | 12.45 ± 1.8 | 14.3 ± 2.3 | 11.5 ± 2.5 |

***L292: Fig. 9***

Thank you, we accepted the recommendation and it is involved in the revised version of the manuscript as “Fig. 9”

***Very importantly; please uniform 4-HPPD or HPPD through the manuscript.***

Thank you, we accepted the recommendation and it is uniformed in the revised version of manuscript as “4-HPPD”.

***Please use “T” as a substitute for testosterone through the manuscript, after expanding the abbreviation in the first position.***

Thank you, we accepted the recommendation and it is substituted to “T” through the manuscripts in the revised version.

***L324: ip >>> Please use “ip” as a substitute for intraperitoneal through the manuscript, after expanding the abbreviation in the first position.***

Thank you, we accepted the recommendation and it is “ip” throughout the manuscript in the revised version

***Please add a data availability statement.***

Thank you, we accepted the recommendation and the data are included in the revised version of the manuscript as follow:

Supporting information

S1 Fig 3- The in vivo effect of solvent on the contraction of rat uterine muscle.

S2 Fig 4- The effect of time passage on the contraction of the rat uterine muscle in vivo.

S3 Fig 5- Plasma T concentration-time curves after a single ip injection (10 mg/kg) in non-pregnant and 22^nd^ day pregnant rats.

S4 Fig 6- Plasma T levels before and 30 min after the single dose of T administration in non-pregnant and 22nd day pregnant rats.

S5 Fig 7- The inhibitory effect of T and nifedipine on non-pregnant and 22^nd^ day pregnant uterine tissues in vitro.

S6 Fig 8- The non-genomic uterine relaxing effect of T in vivo.

S7 Fig 9- Plasma levels of 4-HPPD after T administration.

3- ***Please provide an in-brief description for the representative schismatic of Figure 10. If some pathways in the schematic have been previously investigated, please provide a list of references that support the pathways shown in the schematic.***

Thank you, we accepted the recommendation and the sentences are involved in the revised version of the manuscript as:

**“Schematic diagram summarizing the effect of T on inhibiting uterine contraction.** The red line represents the inhibition demonstrated in our experiment, while the blue (inhibition) and green (activation) lines represent the mechanisms described in previous studies. T inhibits voltage dependent Ca^2+-^channels having a crucial role in its uterine relaxing effect (red line). Furtherly, T stimulates the 7TM (G-protein coupled) receptors (short green arrow) and increases cAMP level of the uterine tissues which inhibits the Ca^2+^channels activity (blue line) (32). Additionally, T activates different K^+^ channel types in smooth muscle (long green arrow), that also leads to relaxation by reducing intracellular K^+^ level (33). AC: Adenylyl cyclase, ATP: adenosine triphosphate, cAMP: cyclic adenosine monophosphate, T: testosterone, 7TM: seven-transmembrane receptor (G-protein coupled receptor)”.

4- ***For showing track changes, please use the Office Platform to compare the past version as a source (in your case R1) and the revised version (in your case, R2). This will help to see all details perfoemed during revision.***

The revised version is trackable now as it was requested.
